# Symptoms and risk factors for long COVID in Tunisian population

**DOI:** 10.1186/s12913-023-09463-y

**Published:** 2023-05-15

**Authors:** Souhir Chelly, Sourour Rouis, Olfa Ezzi, Asma Ammar, Sami Fitouri, Asma Soua, Ines Fathallah, Mansour Njah, Mohamed Mahjoub

**Affiliations:** 1 Infection Prevention and Control department, Farhat Hached university hospital of Sousse, Faculty of Medicine of Sousse, University of Sousse, Sousse, 4000 Tunisia; 2 Department of Community and Family Medicine, Faculty of Medicine of Sousse, University of Sousse, Sousse, Tunisia

**Keywords:** Epidemiology, Infectious diseases, COVID-19, Long COVID, Risk factors

## Abstract

**Background:**

The COVID-19 pandemic has presented various challenges, one of which is the discovery that after the acute episode, around 30% of patients experience persistent symptoms or develop new ones, now known as long COVID. This new disease has significant social and financial impacts. The objective is to determine the prevalence of long COVID in the Tunisian population and identify its predictive factors.

**Methods:**

This was a cross-sectional study conducted among Tunisians who were infected with COVID-19 between March 2020 and February 2022. An online self-administered questionnaire was distributed through social media, radio, and television channels over the course of one month (February 2022). Long COVID was defined as the persistence of existing symptoms or the development of new symptoms within three months after onset, lasting for at least two months, and with no differential diagnosis. We performed univariate and multivariate analyses using binary stepwise logistic regression with a significance level set at 5%.

**Results:**

A total of 1911 patients participated in our study, and the prevalence of long COVID was 46.5%. The two most frequent categories were general and neurological post-COVID syndrome, with a prevalence of 36.7% each. The most commonly observed symptoms were fatigue (63.7%) and memory problems (49.1%). In the multivariate analysis, the predictive factors for long COVID were female gender and age of 60 years or older, while complete anti-COVID vaccination was found to be a protective factor.

**Conclusions:**

Our study found that complete vaccination was a protective factor against long COVID, while female gender and age of 60 years or older were identified as the main risk factors. These findings are consistent with studies conducted on other ethnic groups. However, many aspects of long COVID remain unclear, including its underlying mechanisms, the identification of which could guide the development of potential effective treatments.

## Introduction

Coronavirus disease 2019 (COVID-19) became a pandemic on 2020 [1]. About 80% of cases were mild to moderate, while 5% developed critical illness. As of October 16, 2022, there have been 621 million confirmed cases and 6.5 million deaths reported globally [2]. However, research has shown that more than 30–50% of adult patients continue to experience persistent symptoms or develop new ones at one to two months, and 10–15% still have symptoms at six to eight months after infection [3]. These symptoms can be disabling and diverse, as they may affect a patient’s ability to perform daily activities and household chores. They can be related to several organs, with the most common being fatigue, sleep disorders, psychiatric illness, and dyspnea. Based on current literature, these long COVID symptoms tend to evolve slowly and fluctuate towards improvement. However, a significant proportion of patients have also reported no improvement [3, 4]. Long COVID is not yet well understood, but it has been associated with female gender, respiratory symptoms at onset, and the severity of the illness [5]. On the other hand, the social and financial impacts of long COVID-19 are considerable and must be addressed. Research has shown that long COVID can lead to work absences, reduced work productivity, and increased healthcare utilization, which can strain healthcare systems already burdened by the pandemic. Additionally, long COVID can have mental health implications, with some patients experiencing depression, anxiety, and post-traumatic stress disorder. These implications have the potential to affect the overall wellbeing and resilience of individuals and communities. Therefore, understanding the broader implications of long COVID is critical for developing effective management strategies and providing support for affected individuals [6]. A better understanding of the symptoms, risk factors, and treatment of long COVID is necessary to manage this disease more effectively within healthcare systems [7]. To our knowledge, and after conducting a bibliographic search, no studies have been conducted in North Africa that focus on long COVID. It is important to identify the epidemiological and clinical specificities of long COVID in North Africa compared to other ethnic groups and to determine if there is a genetic predisposition that protects the Tunisian population, as was the case for severe forms and mortality [8, 9]. Additionally, many previous studies have focused either exclusively on inpatients or outpatients, but data on the entire population are scarce. Studies using health data in both populations are needed to elucidate and compare symptoms that are independently associated with the long-term effects of COVID-19. Therefore, the aim of this study is to determine long COVID symptoms and their predictive factors among the Tunisian population.

## Methods

### Study design

This was a cross-sectional study conducted in February 2022 among Tunisian individuals who were infected with COVID-19 between March 2020 and February 2022.

### Study population

The study included all Tunisians who were infected with COVID-19 at least once and agreed to participate in the study. The required sample size was calculated using the formula: n=[(Z_α/2_)^2^ ×p x (1-p)]/i^2^. The prevalence of long COVID-19 varies between 5 and 50% [10]. A proportion (p) of long COVID-19 of 50% was chosen to maximize the sample size [10], a precision (i) of 5%, a risk error (α) of 5% and a loss of 30% due to non-eligible participants (not being a Tunisian, under 18 years of age, ect.) were considered, resulting in a required sample size of at least 501 participants. Individuals under 18 years of age, Tunisians living abroad, and non-Tunisian residents in Tunisia were excluded from the study. Subjects who were infected during the last two months were also excluded to allow for a two-month period for the onset of long COVID symptoms.

### Data collection

We collected data using an online self-administered questionnaire developed with Google Forms, which was available in French and Arabic. The questionnaire was distributed through social media (Facebook), radio, and television channels for one month (February 2022) with weekly reminders, since Facebook is the most popular social media in Tunisia [11]. Respondents were asked about their socio-demographic characteristics, COVID-19 infection history, impact on their health, and post-COVID infection symptoms.

### Data analysis

We used the Statistical Package for Social Sciences (SPSS) version 21.0 for data entry and analysis. Quantitative variables were presented as means ± standard deviations if normally distributed and compared using the t-test. Qualitative variables were presented as frequencies and percentages and compared using the chi-square test. Multivariate logistic regression was used to identify predictive factors of long COVID. We included all variables with a p-value less than or equal to 20% in the univariate analysis. The significance threshold was set at 5%, and the strength of association was estimated by calculating the odds ratio (OR) and its 95% confidence interval.

## Variables’ definitions

### Long COVID

The definition of Long COVID adopted in this study was based on the WHO definition [12]. It refers to the condition that occurs in people with a history of probable or confirmed SARS-CoV-2 infection, usually within three months of the onset of COVID-19, with symptoms and effects that last for at least two months, and cannot be explained by an alternative diagnosis. The symptoms can be categorized into various categories [13, 14], such as general syndrome (Fatigue, fever, sweating, general pain, loss of appetite and/or weight, red eyes, arthralgia, muscle pain and weakness, lower limb edema), neurological syndrome (affecting the central and/or peripheral nervous system, including headaches, cognitive disorders such as memory and concentration disorders, epilepsy, and dizziness), psychiatric syndrome (sleep disturbances, irritability, depression, or anxiety), respiratory syndrome (shortness of breath, difficulty breathing, cough), cardiac syndrome (chest pain, burning sensation, palpitations), digestive syndrome (abdominal discomfort, constipation, diarrhea, vomiting, nausea), ear, nose and throat syndrome (ageusia, anosmia, tinnitus, odynophagia), and dermatological syndrome (hair loss, vesicular maculopapular urticarial lesions).

### Ethics approval and consent to participate

This study was conducted in accordance with the ethical principles of the Declaration of Helsinki and was approved by the Ethical Committee of Farhat Hached University Hospital (Reference of opinion of the committee of medical ethics and research: CER:34-2022). Participation in the study was voluntary, and written informed consent was obtained from each participant after clarification of the study objectives and activities. To ensure anonymity and confidentiality, full names and email addresses were not collected.

## Results

### Descriptive analysis

In total, 1911 participated, out of which 1381 were selected. The mean age was 37.25 ± 9.26, and the majority were females (79.9%) with a sex ratio (M/F) of 0.25. Participants belonged to all 24 Tunisian governorates with the highest number from Tunis governorate (n = 267, 19.3%) followed by Sousse (n = 242, 17.5%), Ariana (n = 135, 9.8%), Monastir (n = 80, 5.8%) and Sfax (n = 66, 4.8%).

The prevalence of post COVID was 45.6% (n = 642) and the prevalence of specific post-COVID syndrome categories were as follows: general (36.7%), neurological (36.6%), psychiatric (32.1%), respiratory (16.8%), cardiological (21.6%), ear, nose and throat (22.7%), dermatological (22.8%), and digestive (4.2%).

Most patients with long COVID were females (85.2%) with a sex ratio (M/F) of 0.17 and aged between 30 and 39 years (44.2%). The symptoms of long COVID were fatigue (63.7%), memory disturbances (49.1%), concentration difficulties (49.1%), hair loss (47.6%), mood swings (40.5%), sleeping disturbances (38.5%), depression (36.3%), anxiety (36%), difficulty finding words (34.3%), irritability (33.5%), arthralgia (31.9%) and headache (31.8%) (Table 1). The majority of long COVID patients (64.48%) sought medical consultation, and their treating physician confirmed the diagnosis of long COVID. In one case the diagnosis of myocarditis and pericarditis post COVID was established.

The vaccination status before infection was 73.2% unvaccinated, 7.7% % partially vaccinated and 19.1% fully vaccinated against COVID-19.

**Table 1:** Long COVID symptoms

Categories	Symptoms	n = 642 (%)
**General symptoms**	Fatigue	409 (63.7)
	Arthralgia	205 (31.9)
	Muscle pains	141 (22.0)
	General pain	140 (21.5)
	Intermittent Fever	16 (2.5)
	Sweating	86 (13.4)
	Chills	74 (11.5)
	Lower limb edema	47 (7.3)
	Anorexia	71 (11.1)
	Weight loss	70 (10.9)
	Red eyes	36 (5.6)
**Neurological symptoms**	Headache	204 (31.8)
	Dizziness	190 (29.6)
	Concentration difficulties	315 (49.0)
	Memory disturbances	393 (61.1)
	Difficulty finding words	220 (34.3)
**Psychiatric symptoms**	Irritability	214 (33.3)
	Depression	233 (36.3)
	Anxiety	232 (36.1)
	Mood swings	260 (40.5)
	Sleeping disturbances	250 (38.9)
**Respiratory symptoms**	Dyspnea	99 (15.4)
	Polypnea	118 (18.2)
	Cough	107 (16.7)
**Cardiological symptoms**	Burning sensation	101 (15.7)
	Tachycardia	161 (25.1)
	Palpitations	176 (27.4)
	Chest pain	104 (16.2)
**Ear, Nose and Throat symptoms**	Rhinorrhea	36 (5.6)
	Anosmia	141 (22.0)
	Ageusia	80 (12.5)
	Odynophagia	36 (5.6)
	Tinnitus	164 (25.5)
**Dermatological symptoms**	Cutaneous lesions	38 (5.9)
	Hair loss	306 (47.7)
**Digestive symptoms**	Nausea	29 (4.5)
	Vomiting	15 (2.3)
	Diarrhea	32 (5.0)
	Epigastralgy	5 (0.8)
**Other symptoms**	Hot flushes	1 (0.2)
	Hallucinations	1 (0.2)
	Ear, eye pain	5 (0.8)
	Hyperglycemia	2 (0.3)
	High blood pressure	2 (0.3)

In the univariate analysis, the predictive factors of long COVID were female sex (p ≤ 10^− 3^, OR = 1.92, CI95%[1.46–2.52]), obesity (p = 0.033, OR = 1.02, CI95% [1.06–1.84]), the number of symptoms recorded in acute COVID > 5 (p = 0.008, OR = 3.05, CI95%[1.33–6.98]), comorbidities (p = 0.002, OR = 1.48, CI95%[1.15–1.91]), history of a respiratory affection (p = 0.046, OR = 1.55, CI95%[1.00-2.40]) and being hospitalized in intensive care (p = 0.010, OR = 7.16, CI95%[1.59–32.16]). Having complete vaccination (p ≤ 10^− 3^, OR = 0.47, CI95%[0.35–0.62] ) was a protective factor (Table 2). The multivariate analysis showed that the predictive factors of long COVID were female gender (p = 0.02, OR = 1.66, CI95%[1.21–2.28]), age ≥ 60 years (p = 0.049, OR = 0.049, CI95%[1.00-5.49]), anosmia during acute COVID (p **≤** 10^− 3^, OR = 2.68, CI95%[1.85–3.87]), diarrhea during acute COVID (p = 0.022, OR = 1.33, CI95%[1.04–1.71]), dyspnea during acute COVID (p **≤** 10^− 3^, OR = 1.72, CI95%[1.27–2.33]) and rash during acute COVID (p = 0.011, OR = 1.95, CI95%[1.16–3.27]). Complete anti-COVID vaccination (p ≤ 10^− 3^, OR = 0.22, CI95%[0.16–0.29]) was identified as a protective factor (Table 3).

**Table 2:** Univariate analysis predictive factors of Long COVID

Factor	Long COVID n(%)		p value	OR CI95%
	Yes	No		
**Gender**				
Male	95(6.9)	185 (13.4)	Reference	Reference
Female	547(39.6)	554(40.1)	**≤10** ^**-3**^	1.92 (1.46-2.52)
**BMI**				
<18,5	17(1.2)	22(1.6)	0.964	0.95 (0.52-1.89)
18,5-25	251(18.2)	320(23.2)	Reference	Reference
25-30	237(17.2)	270(19.5)	0.359	1.11 (0.88 -1.42)
>30	137(9.9)	127(9.2)	**0.033**	1.02 (1.06 -1.84)
**Smoking status**				
No	546(39.5)	593(42.9)	Reference	Reference
Yes	96(7)	146(10.6)	**0.02**	0.71 (0.53-0.94)
**Number of symptoms in acute COVID**				
0	7(0.5)	15(1.1)	Reference	Reference
1-5	134(9.7)	255(18.4)	0.43	1.40 (0.60-3.24)
>5	501(36.3)	469(34)	**0.008**	3.05 (1.33-6.98)
**Severity of acute COVID**				
No Hospitalization	590(42.7)	705(51)	Reference	Reference
Hospitalized in a medical unit	40(2.9)	32(2.4)	0.1	1.49 (0.92-2.40)
Hospitalized in intensive care unit	12(0.9)	2(0.1)	**0.01**	7.16 (1.59-32.16)
**Symptoms in acute COVID**				
Soreness	427(30.9)	437(31.6)	**0.007**	1.35 (1.08-1.68)
Chest pain	215(15.6)	252(18.2)	**≤10** ^**-3**^	1.57 (1.25-1.97)
Anosmia	496(35.9)	439(31.8)	**≤10** ^**-3**^	2.32 (1.83-2.93)
Ageusia	450(32.6)	390(28.2)	**≤10** ^**-3**^	2.09 (1.67-2.61)
Nausea	199(14.4)	147(10.6)	**≤10** ^**-3**^	1.80 (1.41-2.31)
Vomit	131(9.5)	91(6.6)	**≤10** ^**-3**^	1.82 (1.36-2.44)
Diarrhea	226(16.4)	276(20)	**≤10** ^**-3**^	1.71 (1.37-2.13)
Arthralgia	413(29.9)	402(29.1)	**≤10** ^**-3**^	1.51 (1.21-1.87)
Myalgia	301(21.8)	284(20.6)	**0.002**	1.41 (1.14-1.75)
Dyspnea	184(13.3)	115(8.3)	**≤10** ^**-3**^	2.18 (1.67-2.83)
Fatigue	510(36.9)	550(39.8)	**0.028**	1.32 (1.03-1.71)
Rhinorrhea	229(16.6)	312(22.6)	**0.013**	0.75 (0.61-0.94)
Rash	48(3.5)	23(1.7)	**≤10** ^**-3**^	2.51 (1.51-4.18)
**Comorbidities**				
No	472(34.2)	984(71.2)	Reference	Reference
Yes	170(12.3)	253(18.3)	**0.002**	1.48 (1.15-1.91)
**Comorbidity**				
Respiratory affection	50(3.6)	38(2.7)	**0.046**	1.55(1.00-2.40)
**Vaccination status**				
No	507(36.7)	505(36.6)	Reference	Reference
Partially	50(3.6)	56(4)	0.56	0.88 (0.59-1.32)
Complete	85(6.1)	179(13)	**≤10** ^**-3**^	0.47 (0.35-0.62)

## Discussion

To our knowledge, this is the first Tunisian study to determine the prevalence of long COVID in the Tunisian population and its predictive factors. The most frequent long COVID syndrome categories were the general and neurological categories with a prevalence of 36.7% each. The most commonly reported symptoms were fatigue (63.7%) and memory problems (49.1%). In the multivariate analysis, the predictive factors for long COVID were female gender and age ≥ 60 years. Complete anti-COVID vaccination was found to be a protective factor. Our study found a prevalence of long COVID of 46.5%, similar to other studies that suggest it occurs in as many as 30% of people infected [15]. The prevalence can vary from 5 to 50% due to its complex definition [10].

**Table 3:** Multivariate analysis predictive factors of long COVID

Factor	Long COVID n (%)		p value	OR CI95%
	Yes	No		
**Gender**				
Male	95(6.9)	185 (13.4)	Reference	Reference
Female	547(39.6)	554(40.1)	**0.02**	1.66 (1.21-2.28)
**Age**				
<30	125(9)	259(18.7)	Reference	Reference
30-39	290(21)	507(36.7)	0.075	1.32 (0.97-1.79)
40-49	177(12.8)	350(25.3)	0.653	1.08 (0.76-1.53)
50-59	40(2.9)	102(7.4)	0.96	1.01 (0.60-1.71)
≥60	18(1.3)	19(1.4)	**0.049**	2.34 (1.00-5.49)
**Symptoms in acute COVID**				
Anosmia	496(35.9)	439 (31.8)	**≤10** ^**-3**^	0.68 (1.85-3.87)
Diarrhea	226(16.4)	276 (20)	**0.022**	1.33 (1.04-1.71)
Dyspnea	184(13.3)	115 (8.3)	**≤10** ^**-3**^	1.72 (1.27-2.33)
Rash	48(3.5)	23 (1.7)	**0.011**	1.95 (1.16-3.27)
**Vaccination status**				
No	507(36.7)	505(36.6)	Reference	Reference
Partially	50(3.6)	56(4)	0.397	0.83 (0.55-1.25)
Complete	85(6.1)	179(13)	**≤10**^**-3**^	0.22 (0.16-0.29)

Most commonly reported symptoms were fatigue (63,4%), memory disturbances (61,1%), hair loss (47,2%), concentration difficulties (48,6%), mood swings (40,5%). These findings are consistent with the literature [16].

Various sociodemographic and clinical risk factors associated with long COVID have been described in the literature. Women have been found to be at a higher risk than men in most studies [3, 17–19], which is consistent with our findings. However, a few studies have found no gender difference [20, 21]. Hormones may contribute to perpetuating the hyperinflammatory status even after recovery from a disease [22], and females may produce stronger IgG antibodies in the early phase of the disease, potentially leading to a more favorable outcome [23]. However, this may also contribute to perpetuating disease manifestations in females. The statement also suggests that women may be more attuned to their bodies and related distress.

In a British study, age above 70 was found to be associated with a higher risk of reporting long COVID symptoms [19]. Initially, we found no differences between age groups, but in the multivariate analysis, older age above 60 was significantly associated with a higher risk of developing long COVID. In contrast, another British study, after adjusting for baseline covariates, found that older age was associated with a lower risk, with those aged 30–39 years having a 6% lower risk and those aged ≥ 70 years having a 25% lower risk compared to those aged 18–30 years [17]. This discrepancy may be due to vaccination campaigns starting later in North Africa than in European countries, or that anti-COVID vaccination was primarily meant for the elderly. However, older adults are at higher risk of experiencing severe illness due to pre-existing conditions such as diabetes, hypertension, and cardiovascular disease [24].

Being overweight or obese has consistently been associated with an increased risk of persistent symptoms in various studies, similar to our findings in the univariate analysis [17, 25]. The COVID-19 pandemic has already demonstrated a strong correlation between obesity and the severity of the viral disease. This may be due to the prolonged virus-induced inflammatory response and hyperactivated pro-inflammatory cytokines and immune cells, leading to prolonged COVID symptoms [26].

In ten UK longitudinal studies and electronic health records, current smoking was found to lower the risk of long COVID [25], which is consistent with our results in the univariate analysis. However, a study found that smokers and former smokers were at an increased risk of reporting long COVID symptoms, compared to those who had never smoked [17]. Thus, this exposure should be further explored.

Studies have identified a high number of initial symptoms during acute COVID as a predictive factor for long COVID [3, 19, 20]. Furthermore, while some studies have set the number of initial presenting symptoms for long COVID risk at more than five [18, 19], our study validated these findings before adjusting baseline covariates. The initial presenting symptoms that were significantly associated with the development of long COVID in our study were anosmia, diarrhea, dyspnea, and rash.

The association between initial disease severity during acute COVID and long COVID has been studied. The results are varied as most studies did not find any association [27–30], while a few reported that patients who needed invasive mechanical ventilation, intensive care unit admission, or prolonged hospitalization were more likely to experience long-term tissue damage associated with persistent symptoms and, thus, long COVID [31, 32]. In our study, initial severity implying the hospitalization in a medical unit or intensive care was only significantly associated with the occurrence of long COVID in the univariate analysis.

Another important potential risk factor that has been examined by a number of studies is preexisting medical conditions. In fact a wide range of comorbidities, such as asthma or depression have been identified as risk factors for long COVID [3, 17, 19, 25].

Similarly to our findings, high cholesterol levels and diabetes are not associated with long COVID [17, 25].

Respiratory conditions are widely associated with long COVID in the literature, with chronic obstructive pulmonary disease being one of the most incriminated conditions, as described in British study, as well as allergies and asthma [3, 17, 19, 25].

Hypertension was not found to be a predictor of long COVID in our study, but this comorbidity is still controversial [17, 25]. While we found respiratory conditions to be a risk factor for long COVID in the univariate analysis, this association was not found in the multivariate analysis. Psychiatric disorders are also associated with long COVID [5, 17, 25], but this was not found to be the case in our study.

A complete anti-COVID vaccination lowered the risk of long COVID by 0.22 (CI95% [0.16–0.29]) in our study. Similarly, a systematic review suggested that vaccination before COVID could reduce the risk of subsequent long COVID. It seems that two doses of vaccine could be more effective than just one dose. However, the impact of vaccination on people with existing long COVID symptoms is still controversial, with some data showing changes in symptoms and others not [33]. Randomized controlled trials are required to validate the effects of vaccination on long COVID symptoms.

Therefore, the reasons for the ongoing ambiguity in long COVID risk factors may be the use of different definitions of the condition. In this perspective, we highlighted our trials to compare our findings to studies using the WHO definition. Another possibility may be variances in reporting, study design, and participants’ clinical and demographic characteristics, as well as the multifaceted pathophysiology of long COVID [18, 19].

The results of the current study must be interpreted with some limitations in mind. First, the cross-sectional nature of the study did not allow for causal relationships to be reported, but only statistical associations. Secondly, the representativeness of the general population needs to be considered, although our study included all the governorates of Tunisia.

## Conclusion

Our study confirmed that full vaccination provides protection against long COVID, while female gender and age ≥ 60 years were identified as significant risk factors. These findings are consistent with previous studies conducted in diverse ethnic groups. However, further research is needed to understand the underlying mechanisms of long COVID, which is crucial for the development of effective treatments. Overall, our study provides valuable insights into the risk factors associated with long COVID, which could inform public health strategies aimed at preventing and managing this condition.

## Data Availability

Available from the corresponding author on reasonable request.

## Abbreviations

COVID-19: Coronavirus disease 2019
